# Race-Associated EGFR and KRAS Mutation Profiles in Lung Adenocarcinoma

**DOI:** 10.3390/genes17080960

**Published:** 2026-08-16

**Authors:** Lovyanne Vergel de Dios, Catherine Wu, Salique H. Shaham, Manish K. Tripathi

**Affiliations:** 1 School of Medicine, The University of Texas Rio Grande Valley, Edinburg, TX 78541, USA; lovyanne.vergeldedios01@utrgv.edu (L.V.d.D.); catherine.wu01@utrgv.edu (C.W.); 2 South Texas Center of Excellence in Cancer Research, School of Medicine, The University of Texas Rio Grande Valley, McAllen, TX 78540, USA; salique.shaham@utrgv.edu; 3 Medicine and Oncology ISU, School of Medicine, The University of Texas Rio Grande Valley, McAllen, TX 78540, USA

**Keywords:** lung adenocarcinoma, EGFR, KRAS, TP53, racial disparity, smoking status

## Abstract

Background: Lung adenocarcinoma (LUAD) is the most prevalent histologic subtype of non-small cell lung cancer (NSCLC) and exhibits considerable molecular heterogeneity. Among the most clinically significant driver alterations are mutations in EGFR and KRAS, both of which influence treatment selection and oncologic outcomes. The prevalence of these mutations varies by race, yet racial minority populations remain underrepresented in genomic studies. EGFR alterations are more frequently observed in Asian patients, while KRAS mutations predominate in non-Asian cohorts. This study aimed to characterize race-associated differences in driver mutation prevalence among Asian, Black, and White patients with LUAD. Methods: A retrospective secondary cohort analysis was performed using publicly available clinicogenomic data from the Lung Adenocarcinoma Met Organotropism cohort, accessed via cBioPortal, comprising 2653 tumor samples. Patients were stratified by self-reported race into Asian, Black, and White cohorts; cases with missing race data were denoted as either other or unknown. Mutation frequencies for EGFR, KRAS, and TP53 were extracted from OncoPrint cohort study views and compared descriptively across groups. Results: Distinct race-associated differences in driver mutation prevalence were observed. Asian patients exhibited the highest frequency of EGFR alterations (64%), compared with Black (41%) and White (28%) cohorts. In contrast, KRAS mutations were least prevalent in Asian patients (10%) and more frequent in White (33%) and Black (23%) cohorts, indicating an inverse distribution between Asian and non-Asian populations. TP53 mutation prevalence was similar in Asian (52%) and White (53%) cohorts but was notably higher in Black patients (65%). Conclusions: Asian patients with LUAD exhibit a distinct molecular profile characterized by EGFR predominance, with direct implications for eligibility for EGFR-targeted tyrosine kinase inhibitor therapy. Black patients may also benefit from EGFR-based targeted therapies, but lack of large genomic data on Black populations warrants further investigation. White cohorts display a KRAS-dominant mutation pattern, suggesting divergent tumorigenic pathways and the potential need for alternative therapeutic strategies. The elevated TP53 frequency in Black patients remains to be further characterized. These findings support integrating race-associated genomic profiling into precision oncology frameworks to improve treatment selection and reduce disparities in outcomes.

## 1. Introduction

Lung cancer remains the leading cause of cancer-related mortality worldwide, accounting for more than 1.8 million deaths annually [1]. Non-small cell lung cancer (NSCLC) constitutes 80–85% of all lung cancer cases. Lung adenocarcinoma (LUAD) is the most common histologic subtype, representing approximately 40% to 60% of all NSCLC cases [2]. Despite advances in early detection, molecular diagnostics, and targeted therapies, lung adenocarcinoma continues to carry a poor prognosis, particularly among patients presenting with advanced or metastatic disease, which remains the most common presentation. Although survival outcomes for non-small cell lung cancer have improved with the incorporation of biomarker-driven therapy, advanced-stage disease continues to be associated with substantially worse long-term survival compared with localized diseases.

A defining hallmark of LUAD is its marked molecular heterogeneity, driven by oncogenic driver alterations that shape tumor initiation, progression, and response to therapy. Among the most clinically significant somatic mutations are those in the epidermal growth factor receptor (EGFR) and KRAS oncogenes. These genes occupy central roles in receptor tyrosine kinase (RTK) and downstream mitogen-activated protein kinase (MAPK) signaling pathways that regulate cell proliferation, survival, and metastasis. EGFR is a transmembrane RTK with an extracellular EGF-binding domain that modulates apoptosis through the JAK/STAT, PI3K/AKT, and MAPK pathways. Activating EGFR mutations confer sensitivity to tyrosine kinase inhibitors (TKIs), making them highly actionable targets in the management of NSCLC [3]. KRAS, by contrast, is a GTPase that acts downstream of EGFR to regulate cell growth via MAPK signaling. KRAS mutations are strongly associated with smoking-related carcinogenesis, generally confer resistance to EGFR-targeted therapies, and are more prevalent in non-EGFR-driven tumors [4].

EGFR and KRAS were selected as the primary mutations of focus in this study because they are the most prevalent and clinically actionable oncogenic driver alterations in LUAD. These mutations define biologically distinct molecular subtypes with varying relationships with smoking exposure, therapeutic response, and eligibility for targeted treatments. The high prevalence of these mutations and their central roles in precision oncology make them especially relevant biomarkers for evaluating race-associated genomic differences in LUAD. TP53 alterations were also evaluated, as TP53 is the most frequently co-mutated tumor suppressor gene in LUAD and plays a central role in genomic instability, DNA damage response, and tumor progression across multiple molecular subtypes. TP53 mutations are common in both EGFR- and KRAS-driven tumors and provide additional context regarding molecular tumor profiles across racial cohorts [5].

Previous molecular epidemiological studies have shown that the prevalence of driver mutations varies significantly across patient populations. Notably, EGFR mutations occur at substantially higher frequencies in Asian patients with LUAD compared to other racial cohorts, with reported mutation rates ranging from 40% to over 60% in East Asian populations [6]. In contrast, KRAS alterations are more frequently identified in non-Asian populations, particularly among White patients, indicating distinct race-associated molecular phenotypes. These genomic differences are clinically relevant, as they may directly influence treatment selection, eligibility for targeted therapy, and oncologic outcomes.

Although previous studies have characterized EGFR enrichment in Asian populations, racial minority groups, particularly Black patients, remain underrepresented in large genomic datasets and the precision oncology literature. Consequently, comparative analyses of race-associated driver mutation patterns across Asian, Black, and White cohorts remain limited. Biomarker testing has been shown to significantly improve 5-year survival rates in patients receiving targeted therapy compared with those receiving non-targeted therapy [7]. Improved characterization of these differences is essential to reducing disparities in biomarker-driven treatment strategies and advancing precision medicine.

Emerging evidence indicates that molecularly distinct LUAD subtypes may arise through different tumorigenic pathways. EGFR-mutated adenocarcinomas have been associated with stepwise progression from atypical adenomatous hyperplasia (AAH) to adenocarcinoma in situ (AIS), minimally invasive adenocarcinoma (MIA), and ultimately invasive adenocarcinoma, supporting an oncogene-driven progression model [8]. In contrast, KRAS-mutated tumors may follow alternative pathways characterized by smoking-associated mutagenesis, dysregulation of the kinase pathway, and epigenetic alterations [9]. These biological distinctions underscore the importance of investigating whether racial differences in mutation frequencies reflect divergent tumorigenic mechanisms.

**Figure 1:**
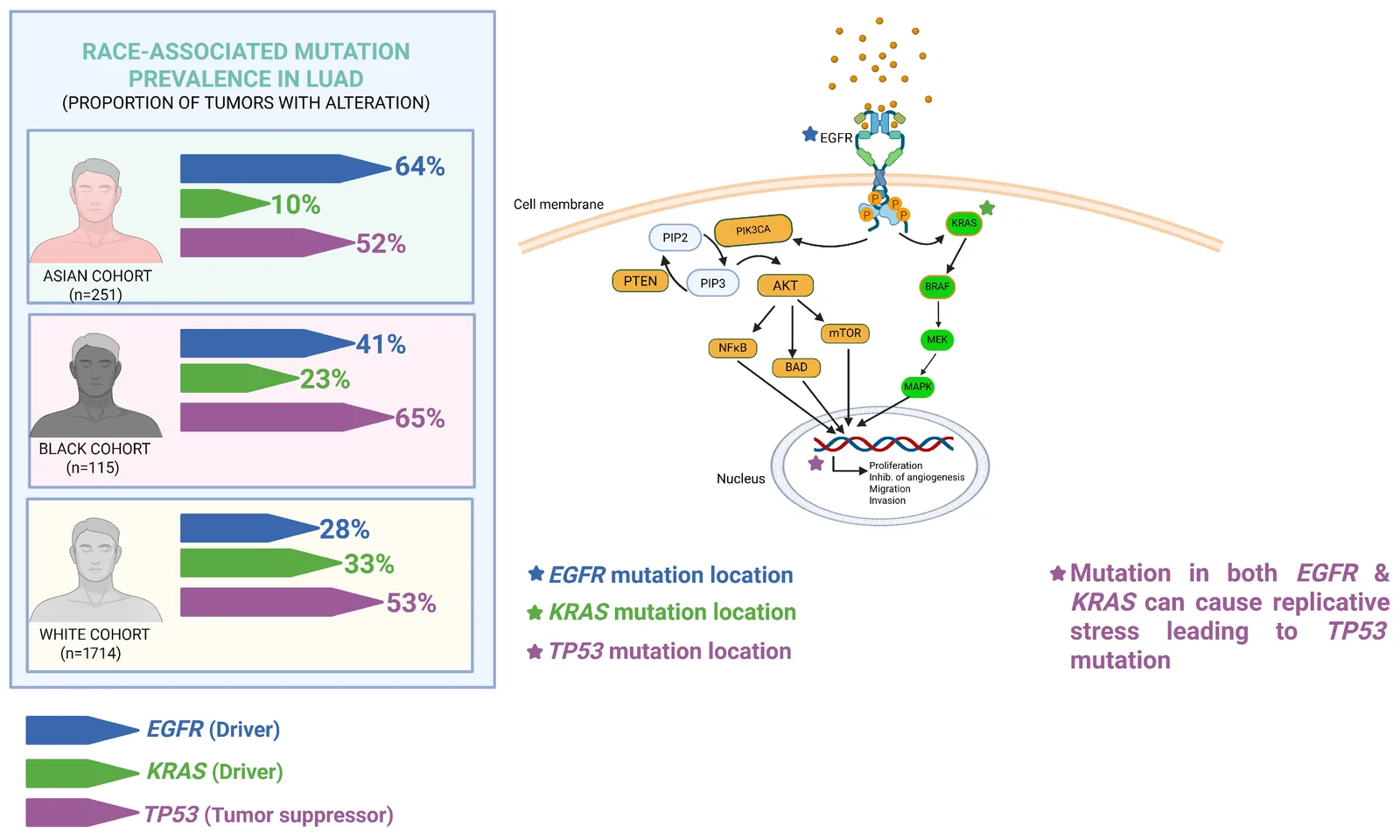
Race-associated prevalence of EGFR, KRAS, and TP53 alterations and their roles in lung adenocarcinoma progression. The schematic depicts major oncogenic signaling pathways associated with EGFR and KRAS activation and TP53 dysfunction.

This study aimed to characterize race-associated differences in driver mutation prevalence in lung adenocarcinoma, with particular emphasis on the predominance of EGFR alterations in Asian and Black patients and the relative prevalence of KRAS mutations in White cohorts. A conceptual overview of the biological roles of EGFR, KRAS, and TP53 alterations and their race-associated prevalence patterns in LUAD is presented in Figure 1.

## 2. Materials and Methods

### 2.1. Study Design and Patients

This study was a retrospective secondary cohort analysis of publicly accessible clinicogenomic data from cBioPortal [10,11,12]. Data were derived from the Lung Adenocarcinoma Met Organotropism cohort (MSK, Cancer Cell 2023), a previously established lung adenocarcinoma (LUAD) sequencing dataset containing 2653 tumor samples from patients with primary and metastatic disease [13].

This cohort was selected because it represents one of the largest publicly available clinicogenomic LUAD datasets with comprehensive molecular profiling, detailed metastatic annotation, smoking-history data, and racial demographic variables. The dataset additionally included both primary and metastatic LUAD tumor samples, enabling characterization of clinically relevant genomic alterations across diverse patient populations.

The parent MSK cohort includes LUAD samples categorized by disease stage, metastatic status, and sequencing timing relative to metastatic progression, including nonmetastatic primary tumors, early-stage tumors that subsequently metastasized, late-stage metastatic primary tumors, metastatic lesions from multiple organ sites, and matched primary–metastatic sample pairs.

For the present analysis, all available LUAD samples in the cBioPortal cohort were queried, and patients were stratified by self-reported race into Asian, Black or African American, and White cohorts. Cases with missing or unknown race information were excluded from comparative analyses.

The primary objective of this study was to evaluate race-associated differences in driver mutation prevalence, with specific emphasis on the predominance of EGFR alterations in Asian patients compared with KRAS-dominant mutations in Black and White cohorts.

**Figure 2:**
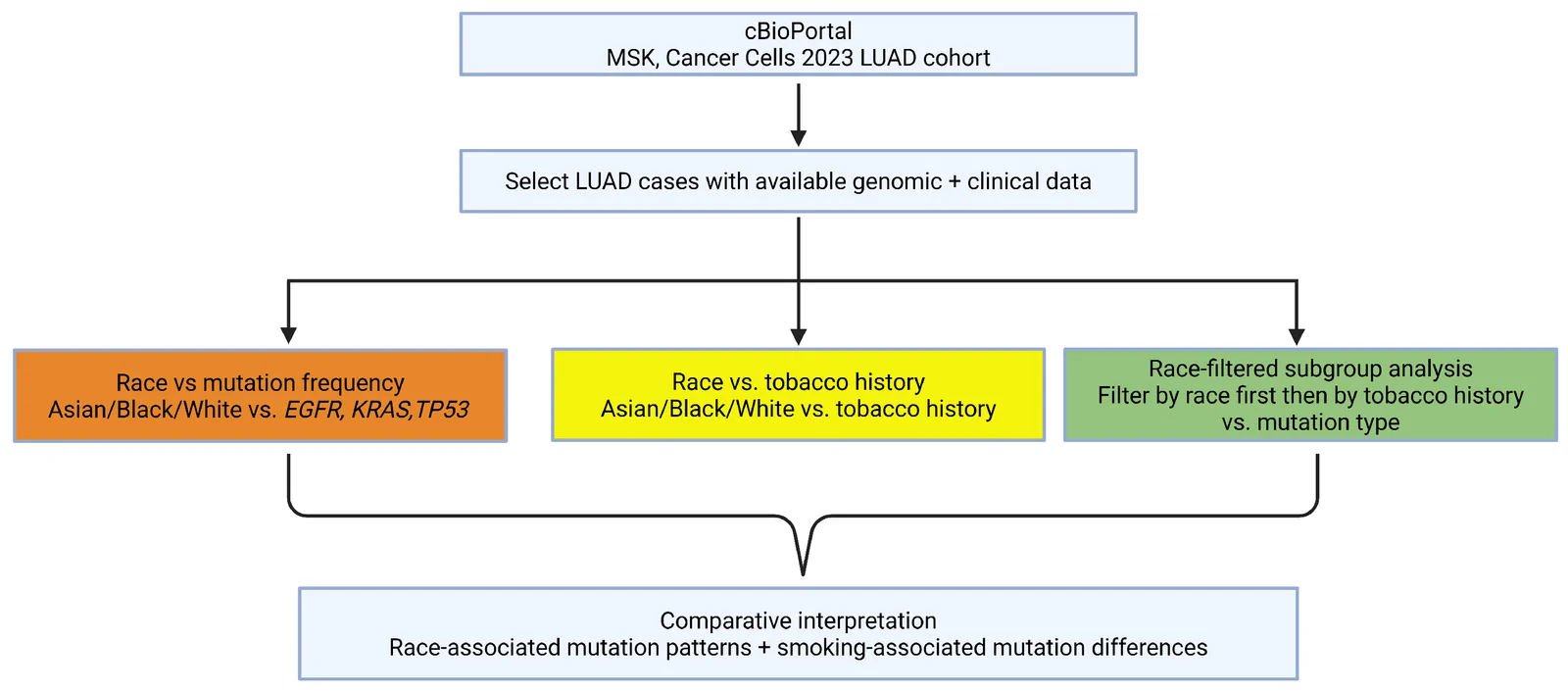
Overview of cohort selection and analytical workflow. LUAD cases from the MSK Lung Adenocarcinoma Met Organotropism cohort were obtained through cBioPortal. Patients were stratified by race and smoking status to evaluate differences in EGFR, KRAS, and TP53 mutation prevalence and to identify race-associated and smoking-associated molecular patterns.

Mutation frequencies for EGFR, KRAS, and TP53 were extracted from race-specific OncoPrint cohort views within cBioPortal. Frequencies were compared descriptively across racial groups, and findings were interpreted in the context of known clinicogenomic disparities in lung adenocarcinoma. The overall analytical workflow used for cohort selection, race stratification, smoking-status analysis, and mutation frequency comparisons is illustrated in Figure 2.

### 2.2. Data Collection

Clinical and genomic data were obtained from the publicly available Lung Adenocarcinoma Met Organotropism cohort (MSK, Cancer Cell 2023) [13] through cBioPortal. Data extraction was performed using the study view and race-specific cohort filters.

Patients were stratified into Asian, Black or African American, and White cohorts using the race category variable available within the study dashboard. For each racial subgroup, genomic alteration frequencies were collected for the primary driver mutations of interest, including EGFR, KRAS, and TP53, using race-filtered OncoPrint cohort views.

Mutation frequencies and percentages were extracted directly from race-specific and smoking-stratified cBioPortal cohort views. Genomic analyses were conducted at the tumor-sample level; therefore, sample-level denominators may exceed patient-level counts because individual patients may contribute more than one tumor sample.

Additional demographic and clinical variables, including age at surgery/biopsy, sex, and smoking status, were collected from the study view dashboard when available. These variables were used to further characterize cohort differences and to evaluate potential clinicogenomic associations.

### 2.3. Statistical Analyses

Mutation frequencies for EGFR, KRAS, and TP53 were reported as percentages within each racial cohort. Comparative analyses were performed descriptively to evaluate differences in mutation prevalence across Asian, Black, and White populations; race category data denoted as other or unknown were excluded from statistical analyses with race stratification. Data visualization and cohort stratification were performed using cBioPortal race-specific OncoPrint and study view tools.

## 3. Results

### 3.1. Patient Demographics

Of a total of 2274 lung adenocarcinoma patients with relevant mutation data, 2080 patients concurrently reported smoking-status information. The cohort included 1714 White, 251 Asian, 115 Black, 97 Other, and 97 patients of unknown race/ethnicity. The average age at surgery/biopsy was 65, and roughly 65% of patients were female (see Table 1 for race-specific breakdown). Among patients with available smoking-history data, the proportion of never-smokers was highest in the Asian cohort (79%), compared with the Black (33%) and White (19%) cohorts. Conversely, the proportion of ever-smokers was highest in the White (81%) and Black (67%) cohorts and lowest in the Asian cohort (21%).

### 3.2. Demographic Factor Differences

The cohort had a relatively small Black patient sample compared with the Asian patient sample. Of the baseline demographics analyzed, only age at surgery/biopsy (*p* < 0.001), never-smoker status (*p* < 0.001), and ever-smoker status (*p* < 0.001) differed significantly across racial groups. Female patients comprised 62–70% of each racial cohort (Table 1). Breakdown by smoking status revealed that, among patients with available smoking-history data, never-smokers predominated in the Asian cohort (79%), whereas ever-smokers predominated in the Black (67%) and White (81%) cohorts (Table 1).

### 3.3. Relationship Between EGFR Mutational Status and Clinical Characteristics

**Figure 3:**
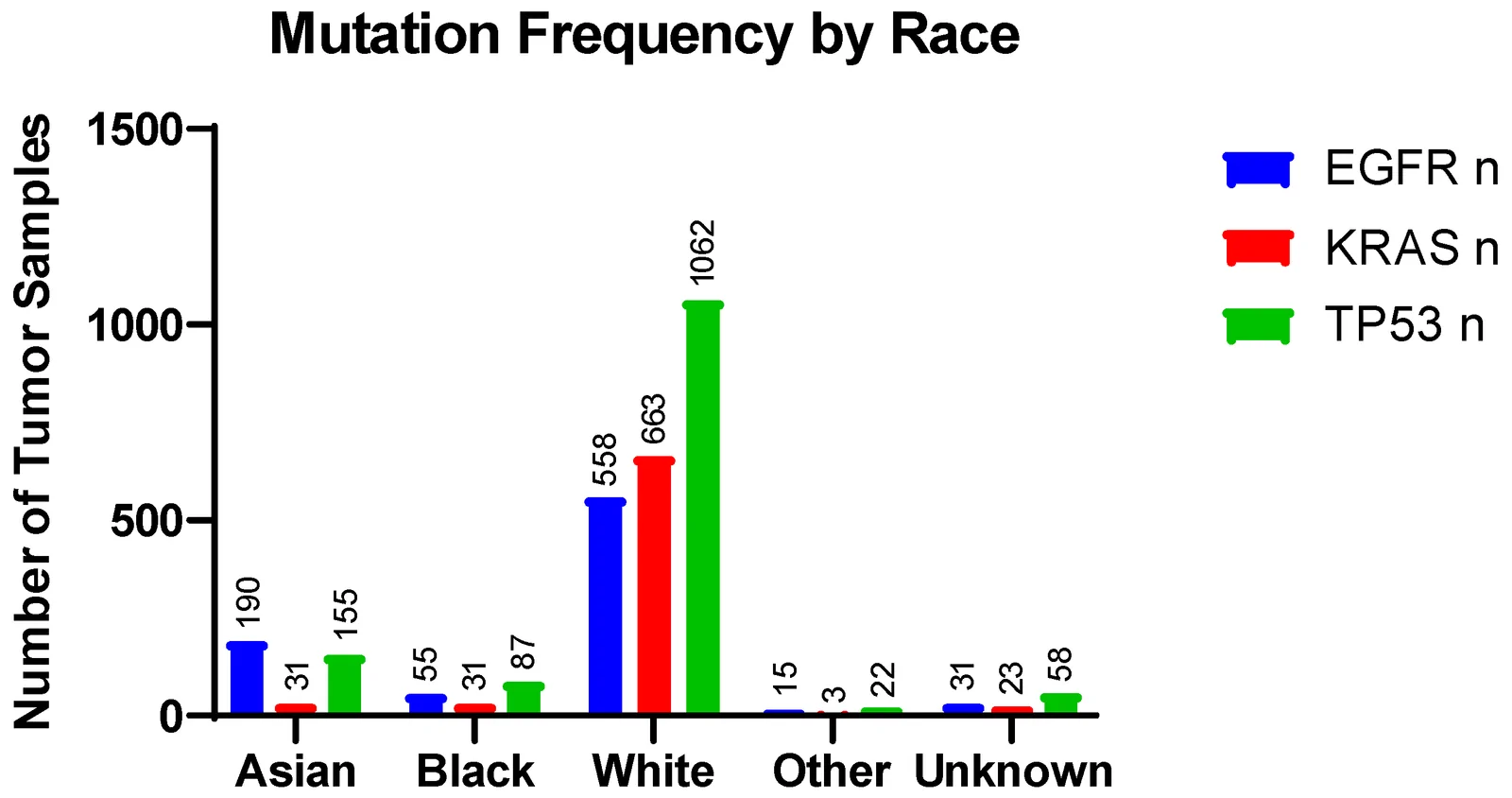
Race-associated frequencies of EGFR, KRAS, and TP53 alterations in lung adenocarcinoma. The number of tumor samples with EGFR, KRAS, and TP53 alterations is shown across racial cohorts. EGFR alterations were comparatively enriched in tumor samples from Asian patients, whereas KRAS alterations were more frequently observed in tumor samples from White patients. These mutation frequency patterns support the existence of race-associated molecular heterogeneity in LUAD.

Tumor samples from Asian patients had the highest frequency of EGFR mutations (64%) and the lowest frequencies of KRAS and TP53 mutations among the racial cohorts analyzed (Table 2). These race-associated mutation patterns are visually illustrated in Figure 3, which demonstrates enrichment of EGFR alterations in Asian patients and greater KRAS mutation frequency among White patients. By smoking status, the EGFR mutation frequency was significantly higher in Asian non-smokers compared with Asian smokers (Table 3). Although the EGFR mutation frequency was relatively high in both Black (41%) and White (28%) patients, TP53 mutation prevalence was higher in both groups (Table 2). Figure 3 further highlights the predominance of TP53 alterations across racial cohorts and the contrasting distribution of EGFR and KRAS mutations. Across all racial subgroups, the EGFR mutation frequency was significantly higher in never-smokers compared to their ever-smoker counterparts, with a less prominent difference in the White cohort (48% vs. 21%) (Table 3).

KRAS mutations were more frequent in tumor samples from Black and White patients than in those from Asian patients. Once stratified by smoking status, KRAS mutation frequencies were significantly higher in ever-smokers among Black (*p* = 0.018), White (*p* < 0.0001), and Asian patients (*p* = 0.005) (Table 3). Lastly, Asian (52%), Black (65%), and White (53%) cohorts demonstrated high TP53 mutation frequencies. TP53 frequency differed significantly by smoking status in the Asian cohort (31% vs. 48%, *p* = 0.016), but not in the Black (57% vs. 52%, *p* = 0.44) or White (37% vs. 46%, *p* = 0.06) cohorts (Table 3).

## 4. Discussion

This study demonstrated notable race-associated differences in the genomic landscape of lung adenocarcinoma, with clear enrichment of EGFR alterations in Asian and Black patients and greater KRAS predominance in White cohorts. Among the three major racial groups analyzed, Asian patients showed the highest prevalence of EGFR mutations (64%), compared with Black (41%) and White (28%) patients. In contrast, KRAS alterations were substantially more frequent in White (33%) and Black (23%) cohorts than in Asian patients (10%). TP53 alterations were common across all groups and showed less pronounced racial variation, occurring in 52% of Asian, 65% of Black, and 53% of White patients. These findings support the existence of distinct race-associated molecular phenotypes in LUAD and underscore the importance of accounting for genomic variation across patient populations when evaluating treatment strategies.

Baseline clinical characteristics further supported these molecular differences. Asian patients demonstrated the highest proportion of never-smokers (79%), compared with Black (33%) and White (19%) cohorts, while ever-smoker prevalence was highest among White patients (81%) and Black patients (67%). These differences were highly statistically significant (*p* < 0.0001). Age at surgery/biopsy also differed significantly across racial groups (*p* < 0.0001), with White patients having the highest median age, followed by Asian and Black patients. In contrast, sex distribution did not significantly differ across cohorts (*p* = 0.30), suggesting that the observed mutation patterns were less likely to be explained by differences in female predominance alone and were more strongly associated with smoking exposure and underlying molecular subtype.

Smoking-stratified mutation analyses provided one of the strongest findings of this study. Within the Asian cohort, EGFR mutations were more common in never-smokers than ever-smokers (70% vs. 37%, *p* < 0.001), while KRAS mutations were less frequent among never-smokers (8% vs. 26%, *p* = 0.005). A similar pattern was observed in Black patients, where EGFR alterations were present in 86% of never-smokers compared with 20% of ever-smokers (*p* = 0.001), whereas KRAS mutations were much lower in never-smokers (7% vs. 44%). In the White cohort, EGFR prevalence was also higher among never-smokers (48% vs. 21%, *p* < 0.0001), while KRAS alterations were reduced in never-smokers (10% vs. 48%, *p* < 0.0001). The consistency of this pattern across all racial groups strongly suggests that smoking history serves as both a clinical predictor and a surrogate marker for distinct genomic subtypes of LUAD.

These findings are consistent with the PIONEER study, which reported EGFR mutation frequencies of 40–60% in East Asian populations and identified ethnicity and smoking status as independent predictors of EGFR mutation frequency, while sex was no longer a significant predictor after stratification by smoking history [6]. Our study extends these observations by directly comparing Asian, Black, and White cohorts within the same clinicogenomic dataset and showing that smoking status modifies mutation prevalence across all racial groups. This is particularly relevant for Black patients, who remain underrepresented in precision oncology datasets despite displaying distinct molecular characteristics. Notably, marked EGFR enrichment in Black never-smokers suggests that smoking exposure may be a stronger biological determinant of EGFR-driven tumorigenesis than race alone, and that racial differences in mutation prevalence may partly reflect differences in smoking rates across populations.

The biological significance of these findings is supported by established molecular pathways in LUAD. EGFR-mutated adenocarcinomas are classically associated with never-smokers, female sex, and adenocarcinomas arising through a stepwise progression model from atypical adenomatous hyperplasia to invasive disease [8]. Activating EGFR mutations drive RTK signaling and confer sensitivity to tyrosine kinase inhibitors, making them among the most clinically actionable genomic alterations in NSCLC [3]. In contrast, KRAS-driven tumors are more strongly linked to tobacco-associated mutagenesis and downstream activation of RAF–MEK–ERK signaling pathways, often demonstrating relative resistance to EGFR-directed therapies [4]. The significantly higher prevalence of KRAS mutations among ever-smokers across all racial cohorts supports the role of tobacco-associated mutational signatures in shaping tumor evolution.

TP53 alterations demonstrated less racial variation and remained highly prevalent across all groups, suggesting that TP53 functions as a broadly conserved tumor suppressor event rather than a race-specific driver mutation. Its relatively stable prevalence across racial cohorts supports its role in genomic instability, DNA damage response, and progression across multiple molecular subtypes of LUAD rather than in defining distinct race-associated molecular phenotypes [5].

These findings have important implications for biomarker-driven treatment strategies and equity in precision oncology. Because EGFR mutation status directly determines eligibility for targeted therapy, failure to recognize race-associated differences in mutation prevalence may contribute to disparities in molecular testing and treatment access. Asian and Black patients and never-smokers may warrant heightened clinical suspicion for EGFR-driven disease, while under-testing of Black patients may lead to missed opportunities for targeted treatment. Improved representation of minority populations in genomic datasets is essential to ensure the equitable implementation of personalized cancer therapy.

Several limitations should be considered. This study was retrospective and relied on publicly available cBioPortal clinicogenomic datasets, which may introduce institutional referral bias and selection bias. Smoking analyses were limited by incomplete clinical metadata and a substantial proportion of unavailable smoking-status records. Broad racial categories prevented more granular analysis of specific Asian ethnic subgroups, which may demonstrate important differences in mutation prevalence. Additionally, treatment response, survival outcomes, and co-mutation analyses were not available within the present dataset and therefore could not be evaluated.

Future studies should incorporate ancestry-based subgroup analyses, co-mutation profiling, and smoking-associated mutational signature analysis to further define the molecular mechanisms underlying race-associated differences in LUAD. Prospective validation in larger and more diverse cohorts may improve biomarker-guided treatment strategies and strengthen precision oncology approaches for historically underrepresented populations.

## Figures and Tables

**Table 1:** Baseline clinical characteristics of lung adenocarcinoma patients stratified by racial cohort. Values are reported as *n* (%) for categorical variables and median (interquartile range [IQR]) for continuous variables. Smoking-status percentages were calculated using only patients with available smoking-history data. Smoking history was available for 91 Asian, 33 Black, and 601 White patients; cases with missing or unavailable smoking data were excluded from percentage calculations. *p*-values were calculated using the chi-squared test for categorical variables and the Kruskal–Wallis test for continuous variables.

Variable	Asian	Black	White	*p*-Value
N	251	115	1714	—
Age at surgery/biopsy, median [IQR]	65.1 (55.2–71.7)	62.6 (55.9–70.4)	67.5 (60.3–74.1)	<0.0001
Female, *n* (%)	155 (62%)	80 (70%)	1072 (63%)	0.30
Never-smoker, *n*/N available (%)	72/91 (79%)	11/33 (33%)	115/601 (19%)	<0.0001
Ever-smoker, *n*/N available (%)	19/91 (21%)	22/33 (67%)	486/601 (81%)	<0.0001

**Table 2:** Driver mutation prevalence by race in lung adenocarcinoma. Values are reported as *n* (%) among tumor samples within each racial cohort. Sample counts may exceed the number of unique patients reported in Table 1 because individual patients may contribute more than one tumor sample. EGFR, KRAS, and TP53 mutation categories are not mutually exclusive, as individual tumor samples may harbor alterations in more than one gene. All race categories and their associated *n* (%) from the Lung Adenocarcinoma Met Organotropism cohort (MSK, Cancer Cell 2023) [13] are reported. Other and unknown racial cohorts were excluded from statistical analyses involving race stratification.

Race Category	Genomic Samples, N	EGFR *n* (%)	KRAS *n* (%)	TP53 *n* (%)
Asian	297	190 (64%)	31 (10%)	155 (52%)
Black	133	55 (41%)	31 (23%)	87 (65%)
White	1988	558 (28%)	663 (33%)	1062 (53%)
Other	31	15 (48%)	3 (10%)	22 (71%)
Unknown	105	31 (30%)	23 (22%)	58 (55%)

**Table 3:** Mutation frequencies are presented as *n* (%) among tumor samples within never-smoker and ever-smoker subgroups for Asian, Black, and White patients with lung adenocarcinoma. The N values represent tumor samples rather than unique patients. Genomic sample counts may exceed the number of unique patients with available smoking-history data because individual patients may contribute more than one tumor sample. The *p*-values represent within-cohort comparisons of the prevalence of EGFR, KRAS, and TP53 mutations using the chi-square test.

Racial Cohort	Smoking Status	Genomic Samples, N	EGFR *n* (%)	KRAS *n* (%)	TP53 *n* (%)	EGFR *p*	KRAS *p*	TP53 *p*
Asian	Never	88	62 (70%)	7 (8%)	27 (31%)	—	—	—
Asian	Ever	27	10 (37%)	7 (26%)	13 (48%)	<0.001	0.005	0.016
Black	Never	14	12 (86%)	1 (7%)	8 (57%)	—	—	—
Black	Ever	25	5 (20%)	11 (44%)	13 (52%)	0.001	0.018	0.44
White	Never	131	63 (48%)	13 (10%)	48 (37%)	—	—	—
White	Ever	549	113 (21%)	461 (48%)	250 (46%)	<0.0001	<0.0001	0.06

## Data Availability

The data analyzed in this study are publicly available from the cBioPortal for Cancer Genomics (https://www.cbioportal.org). Data were derived from the Lung Adenocarcinoma Met Organotropism cohort (MSK, Cancer Cell 2023) [13], which is accessible through the cBioPortal platform.

## Abbreviations

The following abbreviations are used in this manuscript:

LUAD	Lung adenocarcinoma
NSCLC	Non-small cell lung cancer
EGFR	Epidermal growth factor receptor
RTK	Receptor tyrosine kinase
MAPK	Mitogen-activated protein kinase
TKI	Tyrosine kinase inhibitor
